# Comparative Cytotoxic Activity of Wild Harvested Stems and *In Vitro-*Raised Protocorms of *Dendrobium chryseum* Rolfe in Human Cervical Carcinoma and Glioblastoma Cell Lines

**DOI:** 10.1155/2021/8839728

**Published:** 2021-01-06

**Authors:** Bijaya Pant, Pusp Raj Joshi, Sabitri Maharjan, Laxmi Sen Thakuri, Shreeti Pradhan, Sujit Shah, Sven H. Wagner, Basant Pant

**Affiliations:** ^1^Central Department of Botany, Tribhuvan University, Kirtipur, Kathmandu, Nepal; ^2^Annapurna Research Center, Maitighar, Kathmandu, Nepal; ^3^Sails-For-Science Foundation, Roßwein, Germany

## Abstract

From the medicinal orchid *Dendrobium chryseum* Rolfe, which is used in traditional and folk Chinese medicine, the protocorms were raised in Murashige and Skoog (MS) media in three strengths, full strength (FMS), half strength (1/2 MS), and quarter strength (1/4 MS), with or without the phytohormones 6-benzylaminopurine (BAP) and 1-naphthaleneacetic acid (NAA) and coconut water (CW). The comparative cytotoxic activities of the wild and *in vitro*-raised protocorms were evaluated in human cervical carcinoma (HeLa) and human glioblastoma (U251) cell lines by MTT assay. In *in vivo* and *in vitro*, the methanol extracts of *D. chryseum* showed significant cytotoxic activities. Significant growth inhibition (%) and potent IC_50_ values were demonstrated in HeLa cell lines (49.79% (210.5 *μ*g/mL) for *in vitro*-raised *Dendrobium chryseum* (DCT) versus 46.97% (226.5 *μ*g/mL) for wild *Dendrobium chryseum* (DCW)). Similarly, activities against U251 cell lines exhibited also significant inhibition (28.76% (612.54 *μ*g/mL) for DCW and 17.15% (1059.92 *μ*g/mL) for DCT). The cytotoxic activities of both, wild and tissue-cultured samples, were superior in HeLa cells. In U251 cells, the wild sample was more active than the tissue-cultured one with a moderate cytotoxic effect. Hence, protocorm culture may therefore be a promising future tool for producing pharmacologically bioactive compounds in medicinal orchids. Such sustainable technology approach will minimize the pressure on the natural population of threatened but commercially important medicinal orchids.

## 1. Introduction

Orchidaceae is a highly developed and extensively distributed monocotyledonous family consisting of terrestrial, saprophytic, and epiphytic species [1, 2]. Orchids provide a rich source for many natural molecular entities which are used to treat an array of diseases based on a range of effects, such as cell protection due to the presence of antioxidant, antirheumatic, anti-inflammatory, antiviral, diuretic, neuroprotective, antidiabetic, anticancer, and antimicrobial activities [1–16].

*Dendrobium* is the second largest genus of the family Orchidaceae in Nepal. Thirty *Dendrobium* species are found in Nepal, distributed across the tropical, subtropical, and temperate climatic regions [14–17]. *Dendrobium chryseum* is an epiphytic herb found throughout the western and eastern Himalayas at elevations from 1000 to 2150 m. It is popularly used in traditional and folk Chinese medicine for its antipyretic, eye benefiting, and immunomodulatory effects. Yang et al. reported the presence of particular compounds such as bibenzyl-phenanthrene and coumarin derivatives in *D. chryseum*. While the chemical compound isolated from the stem of *D. chryseum* exhibited antioxidant activity [18], polysaccharides from the *D. aurantiacum* var denneanum (*D. chryseum* non Rolfe.) possess tumor-inhibitory and blood glucose-reducing effects *in vivo* [18]. *D. chryseum* Rolfe demonstrated antioxidant properties and an antidiabetic indication in various studies [19]. Its wild resource has been depleted by overexploitation to meet the demand in traditional medicine and the floriculture industry due to its beautiful flowers. Germination of its seeds in nature is very low since seed germination depends on a symbiotic relationship between the seeds and a special mycorrhizal fungus [20–23]. Recent decades have seen a sharp increase in the role of *in vitro* techniques in plant conservation efforts designed to counter the rapid decline in the world's biodiversity [6, 24, 25]. Indeed, *ex situ* cultivation is one of the key elements in modern conservation strategies adopted to address the threats of increasing urbanization, population growth, and industrialization [26]. Furthermore, *in vitro* techniques are highly useful for providing sustainable sources of optimal plant-derived natural products [24–33].

In particular, orchid species have been cultured with various techniques to synthesize bioactive molecules *in vitro* [34, 35]. In the present study, *D. chryseum* was cultured *in vitro* to produce protocorms for the assessment of potential cytotoxic activity and comparison of such activities with its wild counterparts. Protocorm formation is the peculiar stage in orchids in which an embryo transforms and develops into a specific spherical structure with unique characteristics. Protocorms undergo growth and differentiation stages during their development into mature plants [25, 36]. Protocorms contain highly proliferating tissues which accumulate high contents of secondary metabolites [37]. This is the first investigation of the medicinal properties in protocorms of this threatened species *D. chryseum*, especially for its potential as a source for antineoplastic agents. The objectives of the present study were to establish protocorm culture for the production of bioactive secondary metabolites. Subsequently, isolated extract material thereof was examined for its effect between substances originated from protocorms compared to wild plant material in terms of cell proliferation against two representative cancer cell lines: human cervical carcinoma (HeLa) and human glioblastoma (U251), respectively.

## 2. Materials and Methods

### 2.1. Collection of Plant Material

Immature capsules of *D. chryseum*, collected from Godavari, Lalitpur, Central Nepal, at 1579 m above sea level, were used as explants to produce protocorms *in vitro*. The plant was identified by the Nepalese taxonomist Dr. Keshav Rajbhandari as well as from the literature. A voucher specimen of this plant was deposited in the Tribhuvan University Central Herbarium (TUCH) (voucher number P07) in Kathmandu, Nepal. Material from the stems and leaves of the wild plants and the *in vitro* protocorms were subsequently analyzed for their cytotoxic activities.

### 2.2. Sterilization and Inoculation of Seeds

**Figure 1:**
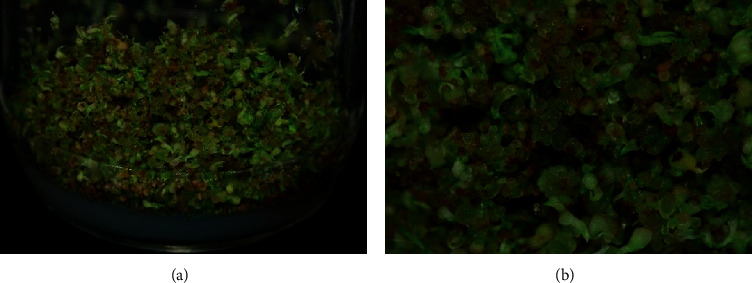
*In vitro*-developed protocorms from germinated immature seeds of *D. chryseum* cultured in 1/2 MS media after 4 months of primary culture. Cultures were maintained under fluorescent light with 8 h dark and 16 h light at 25°C and six replicates were used for each combination.

The procedure for the inoculation of seeds of *D. chryseum* for protocorm formation is already described and reported by us and is followed in the present experiment as well [46]. Immature capsules of *D. chryseum* were first washed under running water added by 2-3 drops of Tween-20 for half an hour to remove dust particles and then rinsed off with distilled water for 10 min. Before inoculation, the explants were surface-sterilized by immersing them first in a solution of 1% sodium hypochlorite and then 70% ethyl alcohol for 2 min each. Finally, they were rinsed off with sterile distilled water at least three times. The surface-sterilized capsules were transferred into Petri dishes and dried on a filter paper in a laminar airflow cabinet. Next, the sterile capsules were cut longitudinally into two equal halves using a sterile surgical blade exposing the seeds within (Figure 1(b)). The seeds were inoculated on an MS medium [38] in three strengths FMS, 1/2MS, and 1/4MS with or without phytohormones (BAP and NAA; on 0.5 mg/L NAA and 1, 1.5, or 2 mg/L BAP) and 10% CW. All cultures were kept in a culture room and maintained at 25 ± 2°C under a photoperiod of 16 h light.

### 2.3. Production of Protocorm Biomass

Protocorms developed from the immature seeds on the different strengths of MS media. All the cultures were maintained for 4 months for the protocorm proliferation which were used for subsequent extraction (Figure 1). The protocorm biomass was separated from the best proliferating medium, and the fresh weight was measured after surface drying. Dry weights were taken after the protocorms were dried at room temperature until mass balance.

### 2.4. Preparation of Plant Material for Extraction

The stems of wild plants and protocorms of *in vitro*-cultured *D. chryseum* were washed in tap water and allowed to completely air-dry in shade. The dried plant materials, the stems and the protocorms, were separately ground into a fine powder and mixed with a solvent in a weight/volume ratio of 1 : 10. Specifically, 20 g of dried powder was dissolved in 200 mL of methanol (Fisher Scientific, ≥99% purity) and placed in a sonicator for extraction. Then, the concentrated plant extract was placed in a rotary evaporator until the solvent was dry and the resultant crude extracts of plant materials were stored in a refrigerator at 4°C for later use.

### 2.5. Evaluation of Cytotoxic Activity of Extracts

The cytotoxic activity of extracts was evaluated in 96-well flat-bottomed microtiter plates (Corning) by using the standard MTT (3-(4,5-dimethylthiazole-2-yl)-2,5-diphenyl-tetrazolium bromide) colorimetric assay with a slight modification. For this purpose, HeLa human cervical carcinoma cells and U251 human glioblastoma cells were cultured in T25 flasks in Eagle's minimum essential medium (EMEM) supplemented with 10% fetal bovine serum (FBS), 1% of penicillin/streptomycin, and 1% L-glutamine and kept at 37°C in a 5% CO_2_ incubator [39]. The cells were seeded in 96-well plates (1 × 10^4^ U251 cells/well and 2 × 10^4^ HeLa cells/well) in 100 *μ*L of the medium and incubated in a 5% CO_2_ incubator at 37°C for 24 h. Following attachment and cell confluence, the cells were treated with different concentrations of the plant extracts (50, 100, 200, and 400 *μ*g/mL) for 48 h. Crude extracts were diluted by dissolving them in 0.1% DMSO (dimethyl sulfoxide, Merck, ≥99.9%) and the EMEM culture medium. The assay was performed in triplicate. Following 48 h of incubation, the supernatant was removed and 150 *μ*L of the medium was combined with 50 *μ*L of MTT for each well. Following 4 h of incubation, a purple formazan crystal was produced. Then, 100 *μ*L of 0.1% DMSO was added and the plates were incubated for a further 15 min at room temperature to dissolve the formazan crystals. The absorbance of samples was measured with a microplate reader (iMark^TM^, Bio-Rad) at a wavelength of 595 nm. A commercially available *cis*-platin drug was used as positive control. The percentage of cytotoxic activity was calculated by the following formula:

(1) $$\begin{array}{c} \%\text{ inhibition }=\;\frac{\text{Ab}1-\text{Ab}2}{\text{Ab}1}\times 100, \end{array}$$

where Ab1 is the absorbance of cells with all components except plant extracts and Ab2 is the absorbance of cells with all components, including plant extracts.

A dose-response curve was plotted for each extract to calculate the 50% inhibition of cell growth (IC_50_ *μ*g/mL of the extract). The cytotoxic capacities of extracts (IC_50_) were calculated using a regression equation.

### 2.6. Statistical Analysis

The average values of a week's measurements were calculated for *in vitro* seed germination and protocorm development. The tests for cytotoxic activities were carried out in triplicate. The values were presented in the form of mean ± SD, and IC_50_ values of the extracts were calculated using second- or third-order polynomial equation regression models in Microsoft Excel 2016.

## 3. Results

### 3.1. *In Vitro* Seed Germination of *Dendrobium chryseum*

Seeds from immature capsules were used as explants for *in vitro* germination. For protocorm development, they were inoculated on different strengths of MS media (FMS, 1/2MS, and 1/4MS), FMS fortified with CW (5% and 10%) and MS medium supplemented with 0.5 mg/L BAP alone and in combination with 0.5 mg/L NAA, 1 mg/L BAP + 0.5 mg/L NAA, 1.5 mg/L BAP + 0.5 mg/L NAA, and 2 mg/L BAP + 0.5 mg/L NAA (Table 1).

All the tested conditions responded to *in vitro* seed germination and protocorm development of *D. chryseum*. The earliest response for *in vitro* seed germination was observed on 1/2 MS medium alone and on FMS medium supplemented with 5% and 10% CW which took three weeks of culture for germination (Table 2). This medium condition was also found to be effective for the earlier development of protocorms which took four weeks of culture incubation (Table 1). However, a large number of protocorms was observed on 1/2 MS medium specified with the highest dry biomass yield of 3.5 g as compared to other tested conditions. Thus, obtained protocorms were used for the phytochemical extraction (Figure 1 and Table 1).

### 3.2. Cytotoxic Activity of *D. chryseum* in HeLa and U251 Cancer Cell Lines

**Figure 2:**
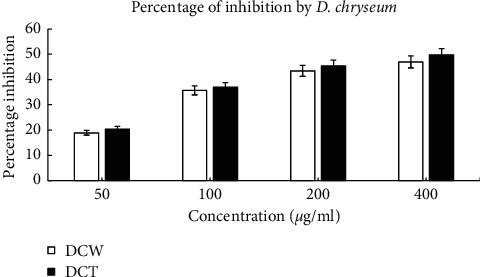
Percentage of inhibition of HeLa cell proliferation by *D. chryseum* derived from DCW and DCT samples.

**Figure 3:**
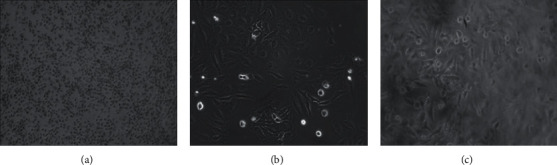
Microscopic images of different stages of HeLa cell proliferation. (a) Confluent HeLa cells; (b) cells grown in a single well; (c) cells after treatment with the extract. Culture condition: cells were cultured in the EMEM at 37°C in a 5% CO_2_ incubator, observed under an Olympus inverted microscope, VImage 2016-US500 camera type.

**Figure 4:**
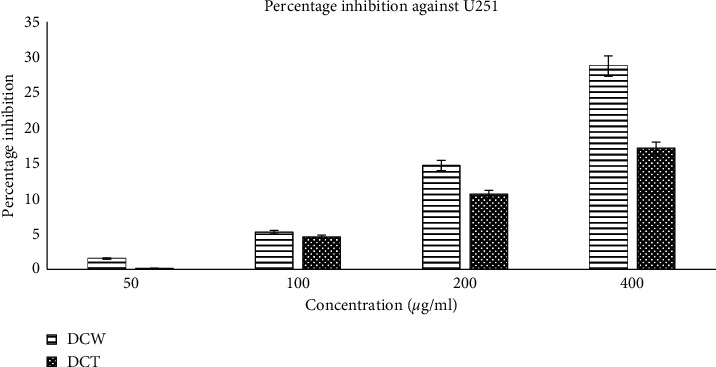
Percentage of inhibition of U251 cell proliferation by *D. chryseum* derived from DCW and DCT samples.

The cytotoxic activities of DCW and DCT were analyzed for their potential roles as antineoplastic agents. The wild samples originated from stem tissue and the *in vitro* samples from protocorms. The methanol extracts of *D. chryseum*, for both wild and *in vitro*-grown samples, showed significant cytotoxic activities (Figures 2–4) for the investigated cancer indications compared to an alkylating standard-of-care cytostatic therapeutic as the control. The results are summarized in Table 3.

Commercially available *cis*-platin had as expected superior potency against HeLa cells with a low IC_50_ value of 25 *μ*g/mL. However, *D. chryseum*-derived samples showed high potency as well through effective inhibition of the growth of HeLa cells. Significant dose-dependent growth inhibition percentages and low IC_50_ values were exhibited: 49.79% (210.5 *μ*g/mL) for DCT and 46.97% (226.5 *μ*g/mL) for DCW while the cytotoxic activities of wild and *in vitro* samples were nearly the same, being slightly better in the *in vitro*-grown samples (Figures 2–3).

*D. chryseum* was found being effective as well in inhibiting U251 cells in a dose-dependent manner with moderate percentages of inhibition and corresponding IC50 values: 28.76% (612.54 *μ*g/mL) for DCW and 17.15% (1059.92 *μ*g/mL) for DCT. The cytotoxic activities of wild and *in vitro* samples differed in such a way that the inhibitory action of samples derived from wild-grown plant material was better than that from *in vitro*-grown samples. However, *in vitro*-grown samples still provided substantial inhibition of the investigated human glioblastoma cell proliferation (Figure 4).

## 4. Discussion

### 4.1. *In Vitro* Seed Germination and Protocorm Development of *Dendrobium chryseum*

In addition to the material *in vivo*, plant cells and tissue culture offer another source of material for rapid plant propagation and the controlled production of essential phytochemicals found in medicinal plants [40–42]. Advances in tissue culture have opened new possibilities for the high-volume production of pharmaceuticals, nutraceuticals, and other beneficial substances. Important medicinal plant species propagated *in vitro* are uniform in secondary and even tertiary metabolites, showing less variation in their contents compared to their wild or cultivated counterparts. Efforts to produce metabolites through tissue culture for various species of plants have been reported by several researchers [6, 43–45].

In the present investigation, seeds of *D. chryseum* were inoculated on the MS basal medium alone and in combination with phytohormones BAP and NAA or with CW as additives. The initiation of seed germination was marked by the formation of green small protocorms. Germination was observed on all MS media. The earlier seed germination was found on 1/2 MS medium alone and FMS medium supplemented with 5% CW and 10% CW within three weeks of culture. Seeds on FMS medium supplemented with BAP (0.5 mg/L) and NAA (0.5 mg/L) took the most time to germinate. The size of protocorms varied depending upon the nature of the nutrient. Small protocorms were observed on 1/4 MS, 1/2 MS, and FMS media with both 5% and 10% CW. These small protocorms developed directly into shoots. In contrast, the protocorms which developed on FMS supplemented with NAA (0.5 mg/L) and BAP (0.5 mg/L) grew quite large by the 24th week but did not develop shoots. Maximum seed germination occurred on 1/2 MS medium which later developed into young protocorms. The result of mass propagation of *D. chryseum* by using protocorm culture is already reported by us [46]. A similar result for the micropropagation of other *Dendrobium* species with a high percentage of germination and protocorm formation included 67% MS [47]. *D. transparens* seeds germinated *in vitro* on a 1/2 MS recipe with 2.0 mg/L of 6-benzyladenine (BA) [47]. Half strength MS medium was effective for the seed germination of both *Vanda dearei* (45%) and *Taprobanea spathulata* (92.73%). Based on the germination result, the present study revealed that all different strengths of MS medium favored seed germination up to the protocorm development. A similar result was also found for the *in vitro* germination of orchid seeds of *Phaius tankervilleae* and *Cymbidium iridioides* [48, 49].

### 4.2. Cytotoxic Activity of Wild and *In Vitro* Protocorms

Previous studies revealed that orchids can be a potent source of anticancer agents. For instance, *D. nobile* showed cytotoxicity against human lung carcinoma, human ovary adenocarcinoma, and human promyelocytic leukemia cell lines [50, 51]. *D. signatum* exhibited cytotoxic activities against human MDA-23 1, HepG2, and HT-29 cancer cell lines [52]. *D. chrysanthum* inhibited the proliferation of leukemia HL-60 cells [11]. *D. longicornu* and *D. moniliforme* showed cytotoxic activities against HeLa and U251 cancer cell lines [4, 5, 35]. *Bulbophyllum kwangtungense* showed antitumor activities against HeLa and K562 human tumor cell lines [53] while *Bulbophyllum odoratissimum* was found to have cytotoxic activities against human cancer cell lines, such as human leukemia cell lines K562 and HL-60, human hepatoma BEL-7402, human lung adenocarcinoma A549, and human stomach cancer cell line SGC-7901 [54]. Prior to this study, no comparative investigation of the cytotoxic activity of plants grown *in vitro* versus wild plants has been reported.

A comparison of the cytotoxic activity of both wild and *in vitro* grown plant samples of *D. chryseum* on HeLa cells revealed that tissue-cultured protocorms (*in vitro* samples) were slightly more active than wild plants. Both, however, showed a significant percentage of growth inhibition and highly potent IC_50_ values in a dose-dependent manner. Upon microscopic investigation, initially cultured HeLa cancer cells before the treatment regimen were elongated and spindle-shaped (Figure 3(b)). Soon after the treatment with the plant extract, treated cells soon reorganised to form condensed round bodies, potentially characterizing the incurring event of apoptosis (cell death) (Figure 4(c)). This cytological study revealed that *D. chryseum* extracts play a pivotal role in inhibiting the growth of HeLa cancer cells, whereas, against the U251 cell line, the activity of the wild sample was comparatively better than the *in vitro*-raised protocorms. Nevertheless, also for this cellular glioblastoma model system, significant percentage of cancer cell proliferation inhibition and corresponding IC_50_ values were demonstrated.

Similarly, a commercially available *cis*-platin drug was found active against HeLa cells and U251 cells with the lowest IC_50_ value and applied as the assay control.

Protocorm suspension cultures have been established for the mass propagation of valuable orchid species [19, 29, 36] and in particular for the production of bioactive compounds including polysaccharides [55]. The presence of bioactive compounds differs depending on the part of the plant and the accumulation of such compounds. It is highly affected by variations in the nutrient components of organic supplements [40, 55, 56]. These studies solidify our claim that *in vitro*-grown protocorms may provide a good source for obtaining bioactive compounds efficiently.

A number of studies about the pharmacological properties of plant metabolites support the findings of this study. The majority of plant-based secondary metabolites are phenols, alkaloids, flavonoids, and tannins [57, 58]. All of these natural products possess diverse pharmacodynamic and pharmacokinetic properties, including cytotoxicity and chemoprevention of carcinogenesis. Flavonoids, triterpenoids, and steroids in particular exert multiple biological effects due to their antioxidant and free radical scavenging mode of action [59]. Further studies have shown particular antioxidant and cytotoxic activities to be associated with a variety of chemical classes of compounds, such as polyphenols, flavonoids, and catechins [60, 61]. A phenol derivative and triterpenes have been isolated from *D. chryseum* [16]. Furthermore, nine chemical compounds, five bibenzyls, three phenanthrenes, and one coumarin derivative have been identified from *D. chryseum*. One of them, isolated from the stem, exhibited antioxidant activity [18]. The *in vitro* propagation of medicinal orchids and the application of other plant tissue culture techniques provide an avenue for the efficient production of selective metabolites, with medicinal use.

## 5. Conclusion

The present findings, based on these first cellular studies, establish the orchid species, *D. chryseum*, as a novel source of active agents with the potential for treating human malignancies. Both wild samples and *in vitro*-grown protocorm mass of *D. chryseum* exhibited cytotoxic activities in a dose-dependent manner against selected cancer cell lines of human cervical carcinoma and human glioblastoma, a type of brain tumor. *In vitro*-cultured protocorms were comparable to wild stems in inhibiting cancer cell proliferation in HeLa and U251 cell lines, respectively. Therefore, protocorm *in vitro* culture offers a highly suitable process for the production of the investigated pharmacologically useful compounds contained in protocorm samples. Further work on the isolation and pharmacological validation against a range of targets and medical indications of bioactive compounds derived from protocorms is needed. However, this may underscore the general attractive utility that protocorms are a potential source for the commercial production of desirable metabolites from *in vitro* culture. Such technology implementation would certainly augment to vast extent very much needed efforts to minimize the pressure on the natural population and their entire habitats of threatened but commercially important medicinal orchids in Nepal.

## Figures and Tables

**Table 1:** Growth response of *Dendrobium chryseum* protocorms from seed culture to MS medium strengths in the presence or absence of phytohormones BAP, NAA, and CW.

Medium composition	Plant growth regulators (mg/L)	Fresh biomass (g)	Dry biomass (g)
FMS	—	13.49	2.28
1/2 MS	—	18.92	3.50
FMS	2 BAP + 0.5 NAA	12.39	1.78
FMS	10% CW	15.34	3
FMS	5% CW	10.34	1.50

**Table 2:** *In vitro* seed germination of *Dendrobium chryseum*.

S. no.	Strength of the MS medium	Plant growth regulators (mg/L) or additives	Observed in weeks	
			Initiation of seed germination	Initiation of protocorm formation
1.	FMS	—	3	5
2.	1/2 MS	—	3	4
3.	1/4 MS	—	3	5
4.	FMS	0.5 BAP + 0.5 NAA	4	7
5.	FMS	1 BAP + 0.5 NAA	4	6
6.	FMS	1.5 BAP + 0.5 NAA	4	6
7.	FMS	2 BAP + 0.5 NAA	3	5
8.	FMS	10% CW	3	4
9.	FMS	5% CW	3	4

Culture condition: cultures were maintained under fluorescent light with 8 h dark and 16 h light at 25°C and six replicates were used for each combination.

**Table 3:** IC_50_ values of the *D. chryseum* extract and *cis*-platin against HeLa and U251 cancer cell lines.

Cell line	HeLa (cervical carcinoma)		U251 (glioblastoma)	
IC_50_	Wild (DCW)	*In vitro* (DCT)	Wild (DCW)	*In vitro* (DCT)
IC_50_ of the extract (*μ*g/mL)	226.5	210.5	612.54	1059.92
IC_50_ of *cis*-platin (*μ*g/mL)	25.00		25.00	

## Data Availability

The data used to support the findings of this study are included within the article.

## References

[1] Stewart S. L., Kane M. E. (2006). Asymbiotic seed germination and *in vitro* seedling development of *Habenaria macroceratitis* (Orchidaceae), a rare Florida terrestrial orchid. *Plant Cell, Tissue and Organ Culture*.

[2] Khasim S., Rao P. M. (1999). Medicinal importance of orchids. *The Botanical*.

[3] Chand M. B., Paudel M. R., Pant B. (2016). The antioxidant activity of selected wild orchids of Nepal. *Journal of Coastal Life Medicine*.

[4] Paudel M. R., Chand M. B., Pant B. (2018). Antioxidant and cytotoxic activities of *Dendrobium moniliforme* extracts and the detection of related compounds by GC-MS. *BMC Complementary Alternative Medicine*.

[5] Paudel M. R., Chand M. B., Pant B. (2017). Cytotoxic activity of antioxidant-rich *Dendrobium longicornu*. *Journal of Pharmacogncy*.

[6] Pant B. (2013). Medicinal orchids and their uses: tissue culture a potential alternative for conservation. *African Journal of Plant Science*.

[7] Rathee P., Chaudhary H., Rathee S., Rathee D., Kumar V., Kohli K. (2009). Mechanism of action of flavonoids as anti-inflammatory agents: a review. *Inflammation & Allergy-Drug Targets*.

[8] Shimura H., Matsuura M., Takada N., Koda Y. (2007). An antifungal compound involved in symbiotic germination of *Cypripedium macranthos* var. rebunense (Orchidaceae). *Phytochemistry*.

[9] Watanabe K., Tanaka R., Sakurai H. (2007). Structure of cymbidine A, a monomeric peptidoglycan-related compound with hypotensive and diuretic activities, isolated from a higher plant, *Cymbidium goeringii* (Orchidaceae). *Chemical & Pharmaceutical Bulletin*.

[10] Iguchi C., Liu Q., Halaweish F., Shao B., Ye Y., Zhao W. (2003). Copacamphane, picrotoxane, and alloaromadendrane sesquiterpene glycosides and phenolic glycosides from *Dendrobium moniliforme*. *Journal of Natural Products*.

[11] Li Y., Wang H., Liu G. (2001). Erianin induces apoptosis in human leukemia HL-60 cells. *Acta Pharmacologica Sinica*.

[12] Suresh K. P., Subramoniam A., Pushpangadan P. (2000). Aphrodisiac activity of *Vanda tessellata* (Roxb.) Hook. ex don extract in male mice. *Indian Journal of Pharmacology*.

[13] Gutiérrez R. M. P. (2010). Orchids: a review of uses in traditional medicine, its phytochemistry and pharmacology. *Journal of Medicinal Plant Research*.

[14] Pant B., Raskoti B. B. (2013). *Medicinal Orchids of Nepal*.

[15] Joshi P. R., Paudel M. R., Chand M. B. (2020). Cytotoxic effect of selected wild orchids on two different human cancer cell lines. *Heliyon*.

[16] Yang L., Wang Z., Xu L. (2006). Phenols and a triterpene from *Dendrobium aurantiacum* var. denneanum (Orchidaceae). *Biochemical Systematics and Ecology*.

[17] Rajbhandari K. R. (2015). *A Handbook of the Orchids of Nepal*.

[18] Yang L., Han H., Nakamura N., Hattori M., Wang Z., Xu L. (2007). Bio-guided isolation of antioxidants from the stems of *Dendrobium aurantiacum* var. denneanum. *Phytotherapy Research*.

[19] Young P. S., Murthy H., Yoeup P. K. (2000). Mass multiplication of protocorm-like bodies using bioreactor system and subsequent plant regeneration in Phalaenopsis. *Plant Cell, Tissue Organ Culture*.

[20] Liu H., Luo Y., Liu H. (2010). Studies of mycorrhizal fungi of Chinese orchids and their role in orchid conservation in China—A review. *The Botanical Review*.

[21] Pant B., Pradhan S., Paudel M. Various culture techniques for the mass propagation of medicinal orchids from Nepal.

[22] Shah S., Shrestha R., Maharjan S., Selosse M.-A., Pant B. (2019). Isolation and characterization of plant growth-promoting endophytic fungi from the roots of *Dendrobium moniliforme*. *Plants*.

[23] Chand K., Shah S., Sharma J., Paudel M. R., Pant B. (2020). Isolation, characterization, and plant growth-promoting activities of endophytic fungi from a wild orchid *Vanda cristata*. *Plant Signaling & Behavior*.

[24] Regmi T., Pradhan S., Pant B. (2010). *In vitro* mass propagation of an epiphytic orchid, *Cymbidium aloifolium* (L.) Sw., through protocorm culture. *Biotechnology Research International*.

[25] Pradhan S., Tiruwa B., Subedee B. R., Pant B. (2014). *In vitro* germination and propagation of a threatened medicinal orchid, *Cymbidium aloifolium* (L.) Sw. through artificial seed. *Asian Pacific Journal of Tropical Biomedicine*.

[26] Pfab M. F., Scholes M. A. (2004). Is the collection of Aloe peglerae from the wild sustainable? An evaluation using stochastic population modelling. *Biological Conservation*.

[27] Shrestha U. K., Pant B. (2012). Production of bergenin, an active chemical constituent in the callus of *Bergenia ciliata* (Haw.) Sternb. *Botanica Orientalis: Journal of Plant Science*.

[28] Adhikari S. R., Pant B., Pokhrel K. (2012). Antimicrobial activity of chemical compounds from *in vivo* roots and *in vitro* callus of *Withania somnifera* (L.) Dunal. *Biomedicine and Biotechnology*.

[29] Ochoa-Villarreal M., Howat S., Hong S. (2016). Plant cell culture strategies for the production of natural products. *BMB Reports*.

[30] Espinosa-Leal C. A., Puente-Garza C. A., García-Lara S. (2018). *In vitro* plant tissue culture: means for production of biological active compounds. *Planta*.

[31] Hussain M. S., Rahman M. A., Fareed S., Ansari S., Ahmad I., Saeed M. (2012). Current approaches toward production of secondary plant metabolites. *Journal of Pharmacy and Bioallied Sciences*.

[32] Vanisree M., Lee C.-Y., Lo S.-F. (2004). Studies on the production of some important secondary metabolites from medicinal plants by plant tissue cultures. *Botanical Bulletin Academia Sinica*.

[33] Bourgaud F., Gravot A., Milesi S., Gontier E. (2001). Production of plant secondary metabolites: a historical perspective. *Plant Science*.

[34] Yoon Y.-J., Murthy H. N., Hahn E. J., Paek K. Y. (2007). Biomass production ofAnoectochilus formosanus hayata in a bioreactor system. *Journal of Plant Biology*.

[35] Paudel M. R., Chand M. B., Pant B., Pant B. (2019). Assessment of antioxidant and cytotoxic activities of extracts of *Dendrobium crepidatum*. *Biomolecules*.

[36] Regmi T., Pradhan S., Pant B. (2017). *In vitro* mass propagation of an epiphytic orchid, *Cymbidium aloifolium* (L.) Sw., through protocorm culture. *Biotechnology Journal International*.

[37] Wang H.-Q., Jin M.-Y., Paek K.-Y., Piao X.-C., Lian M.-L. (2016). An efficient strategy for enhancement of bioactive compounds by protocorm-like body culture of *Dendrobium candidum*. *Industrial Crops and Products*.

[38] Murashige T., Skoog F. (1962). A revised medium for rapid growth and bio assays with tobacco tissue cultures. *Physiologia Plantarum*.

[39] Mosmann T. (1983). Rapid colorimetric assay for cellular growth and survival: application to proliferation and cytotoxicity assays. *Journal of Immunological Methods*.

[40] Pant B. (2014). Application of plant cell and tissue culture for the production of phytochemicals in medicinal plants. *Infectious Diseases and Nanomedicine II*.

[41] Park S.-Y., Ho T.-T., Paek K.-Y. (2020). Medicinal orchids: production of bioactive compounds and biomass. *Orchid Biology: Recent Trends & Challenges*.

[42] Murthy H. N., Paek K.-Y., Park S.-Y. (2018). *Micropropagation of Orchids by Using Bioreactor Technology, Orchid Propagation: From Laboratories to Greenhouses—Methods and Protocols*.

[43] Yue W., Ming Q.-l., Lin B. (2016). Medicinal plant cell suspension cultures: pharmaceutical applications and high-yielding strategies for the desired secondary metabolites. *Critical Reviews in Biotechnology*.

[44] Murthy H. N., Lee E.-J., Paek K.-Y. (2014). Production of secondary metabolites from cell and organ cultures: strategies and approaches for biomass improvement and metabolite accumulation. *Plant Cell, Tissue and Organ Culture (PCTOC)*.

[45] Naing A. H., Chung J. D., Park I. S. (2011). Efficient plant regeneration of the endangered medicinal orchid, *Coelogyne cristata* using protocorm-like bodies. *Acta Physiologiae Plantarum*.

[46] Lim S., Thakuri L. S., Thapa B. B. (2020). *In vitro* propagation of the endangered orchid *Dendrobium chryseum* Rolfe from protocorms culture. *Nepal Journal of Science and Technology*.

[47] Da Silva J. A. T., Cardoso J. C., Dobránszki J. (2015). Dendrobium micropropagation: a review. *Plant Cell Report*.

[48] Sunitibala H., Kishor R. (2009). Micropropagation of *Dendrobium transparens* L. from axenic pseudobulb segments. *Indian Journal of Biotechnology*.

[49] Pant B., Shrestha S., Pradhan S. (2011). *In vitro* seed germination and seedling development of *Phaius tancarvilleae* (L’Her.) Blume. *Scientific World*.

[50] Pant P., Swar S. (2011). Micropropagation of *Cymbidium iridioides*. *Nepal Journal of Science and Technol*.

[51] Lee Y., Park J., Beak N., Kim S., Ahn B. (1995). *In vitro* and *in vivo* Antitumoral phenanthrenes from the aerial parts of *Dendrobium nobile*. *Planta Medica*.

[52] Zhou X.-M., Zheng C.-J., Gan L.-S. (2016). Bioactive phenanthrene and bibenzyl derivatives from the stems of *Dendrobium nobile*. *Journal of Natural Products*.

[53] Chen A., Muangnoi C., Likhitwitayawuid K. (2016). A new bibenzyl-phenanthrene derivative from *Dendrobium signatum* and its cytotoxic activity. *Nature Product Communcation*.

[54] Paudel M. R., Joshi P. R., Chand K. (2020). Antioxidant, anticancer and antimicrobial effects of *in vitro* developed protocorms of *Dendrobium longicornu*. *Biotechnology Reports*.

[55] Wu B., He S., Pan Y.-j. (2006). New dihydrodibenzoxepins from *Bulbophyllum kwangtungense*. *Planta Medica*.

[56] Chen Y., Xu J., Yu H. (2007). 3,7-Dihydroxy-2,4,6-trimethoxyphenanthrene, a new phenanthrene from *Bulbophyllum odoratissimum*. *Journal of Korean Chemical Society*.

[57] Zha X.-Q., Luo J.-P., Jiang S.-T., Wang J.-H. (2007). Enhancement of polysaccharide production in suspension cultures of protocorm-like bodies from *Dendrobium huoshanense* by optimization of medium compositions and feeding of sucrose. *Process Biochemistry*.

[58] Cui H.-Y., Murthy H. N., Moh S. H., Cui Y.-Y., Paek K.-Y. (2015). Establishment of protocorm suspension cultures of *Dendrobium candidum* for the production of bioactive compounds. *Horticulture, Environment, and Biotechnology*.

[59] Ng T. B., Liu J., Wong J. H. (2012). Review of research on Dendrobium, a prized folk medicine. *Applied Microbiology and Biotechnology*.

[60] Gupta M., Mazumder U. K., Kumar R. S., Sivakumar T., Vamsi M. L. M. (2004). Antitumor activity and antioxidant status of *Caesalpinia bonducella* against Ehrlich ascites carcinoma in Swiss albino mice. *Journal of Pharmacological Sciences*.

[61] Uddin S. J., Grice I. D., Tiralongo E. (2011). Cytotoxic effects of Bangladeshi medicinal plant extracts. *Evidence Based Complementary Alternative Medicine*.

